# A low level of serum total testosterone is independently associated with nonalcoholic fatty liver disease

**DOI:** 10.1186/1471-230X-12-69

**Published:** 2012-06-12

**Authors:** Sunmi Kim, Hyuktae Kwon, Jin-Ho Park, Belong Cho, Donghee Kim, Seung-Won Oh, Cheol Min Lee, Ho-Chun Choi

**Affiliations:** 1 Department of Family Medicine, Kangwon National University Hospital, Kangwondo, South Korea; 2 Department of Family Medicine, Healthcare Research Institute, Seoul National University Hospital Healthcare System Gangnam Center, Seoul, South Korea; 3 Department of Family Medicine, Seoul National University Hospital, Seoul National University College of Medicine, Seoul, South Korea; 4 Department of Internal Medicine, Healthcare Research Institute, Seoul National University Hospital Healthcare System Gangnam Center, Seoul, South Korea

**Keywords:** Nonalcoholic fatty liver disease, Total testosterone, Visceral adipose tissue

## Abstract

**Background:**

The association between low serum testosterone levels, visceral adipose tissue (VAT), and metabolic syndrome is now well known. However, the relationship between hepatic steatosis and serum testosterone levels has not been extensively studied. Our aim was to investigate the association of serum total testosterone levels with nonalcoholic fatty liver disease (NAFLD), adjusting for the influence of VAT and insulin resistance.

**Methods:**

This study is a retrospective observational cross-sectional one of healthy Korean men and was conducted at the Seoul National University Hospital Healthcare System Gangnam Center. We used data obtained from 495 men who were at least 20 years of age and who had undergone blood testing, abdominal computed tomography, and ultrasonography. Multiple logistic regression analysis was used to explore the association of serum total testosterone levels with NAFLD.

**Results:**

Men in the low serum testosterone quintile were at a higher risk for NAFLD than men in the highest serum testosterone quintile. After adjusting for age, smoking, diabetes, exercise, BMI, triglycerides, and high-density-lipoprotein cholesterol, subjects with serum testosterone levels in the lowest quintile had an odds ratio (OR) (95% confidence interval (CI)) of 5.12 (2.43–10.77) for NAFLD (*p* value, 0.0004). The inverse association between serum testosterone and NAFLD was attenuated by further adjustment for variables including VAT; however, it remained statistically significant (OR (95% CI): 4.52 (2.09–9.80) in the lowest quintile; *p* value=0.004).

**Conclusions:**

A low serum total testosterone level was independently associated with NAFLD. This report is the first one suggesting the association remains unchanged even after controlling for VAT and insulin resistance.

## Background

Nonalcoholic fatty liver disease (NAFLD) is one of the most common causes of chronic liver disease in South Korea [1] as well as in North America and Europe, where the prevalence of NAFLD is reported to be up to 30% in the general population [2,3]. NAFLD has been found to be closely related to abdominal obesity, dyslipidemia, type 2 diabetes, and metabolic syndrome, all of which are risk factors for cardiovascular disease (CVD) [4-6]. Furthermore, several recently published prospective studies have shown that NAFLD is an independent risk factor for CVD [7-11]. Insulin resistance is regarded as the major factor contributing to the pathogenesis of NAFLD [12]; moreover, NAFLD has been reported to be a very early and sensitive indicator of insulin resistance [13,14]. Similarly, increased visceral adipose tissue (VAT), which induces insulin resistance and plays a key role in the development of metabolic syndrome, has been well established as a major risk factor for NAFLD [15,16].

Numerous studies have shown that low serum testosterone levels in men are associated with an increased risk of obesity, abdominal obesity, and metabolic syndrome [17-20]. The inverse association between serum testosterone levels and metabolic syndrome is often explained by VAT accumulation and insulin resistance [19,21-23]. Testosterone exerts contrasting influences on muscle and VAT by affecting the commitment of pluripotent stem cells and by suppressing the growth of preadipocytes [19]. Based on the inverse correlation of VAT, metabolic syndrome, and insulin resistance to serum testosterone levels [19,21-23], one expects a similar relation between hepatic steatosis and serum testosterone levels; however, this potential relationship has not been extensively investigated. In an early study, 10 patients with hepatic steatosis were reported to have lower serum testosterone levels than reference subjects of the same age [24]. A German study found that men with low serum testosterone levels were at a higher risk of hepatic steatosis than men with high serum testosterone levels [25]. Recently, a study that investigated a cohort of 117 men showed that raising serum testosterone concentrations to normal levels by treatment with parenteral testosterone for 1 year improved the levels of alanine aminotransferase (ALT) and aspartate aminotransferase (AST), as well as weight, body mass index (BMI), waist size, and lipid profiles [26]. Low serum testosterone levels are thus closely related with VAT accumulation and insulin resistance [17,22], both of which are known to play important roles in the pathogenesis and prognosis of NAFLD [15]. We hypothesized that the testosterone level would be associated with NAFLD and that VAT might have a confounding effect on their association. In the present study, our aim was to investigate the association of serum total testosterone levels with NAFLD after adjusting for the influence of VAT and insulin resistance.

## Methods

### Participants and study design

This study is a retrospective observational cross-sectional one of healthy Korean men who were aged 20 years or more and who voluntarily visited the Seoul National University Hospital Healthcare System Gangnam Center between January 2008 and April 2010 for routine health checkups. Study data were obtained using questionnaires, anthropometric measurements, blood samples, abdominal computed tomography (CT), and ultrasonography. Among 28,670 examinees, those with missing data on key variables (1,298 examinees who had not undergone abdominal ultrasonography; 21,425, abdominal CT scans; 4890, serum total testosterone determination) were excluded from the study. We included only the remaining 1057 men who underwent an examination of serum total testosterone, abdominal ultrasonography, and abdominal CT scans of their own accord, which was necessarily a convenience sample. All examinees routinely took serology tests for hepatitis B surface antigen (HBsAg) and hepatitis C antibody (anti-HCV). Of the remaining 1057 men, 77 subjects were excluded on the grounds of HBsAg or anti-HCV positivity. By using self-report questionnaires, we also excluded 433 men who consumed more than 140 g of alcohol per week, had a medical history of other types of hepatitis such as autoimmune hepatitis and chronic viral liver disease, cholestasis, and other metabolic liver diseases, or had previously used steatogenic medications including antiretroviral drugs, antiarrhythmic drugs, anticancer drugs, corticosteroids and hormone.

In addition, 52 subjects were excluded because of missing data on key variables such as smoking, medical history of diabetes, and physical activity. The final analysis included 495 men. Among the participants included, there were no subjects who had the evidence of hepatic cirrhosis or portal hypertension (such as splenomegaly and esophageal varix) on ultrasonography, abdominal CT or gastroscopy. This study protocol was approved by the Seoul National University Hospital Institutional Review Board.

## Measurements

### Abdominal ultrasonography

The diagnosis of NAFLD was based on abdominal ultrasonography (Acuson, Sequoia 512; Siemens, Mountain View, CA) performed by experienced radiologists blinded to the medical information and the laboratory parameters of the participants. Fatty infiltration of the liver was diagnosed if hepatorenal contrast and liver brightness were detected [27]. The subjects thus identified as having a fatty liver in the absence of other potential causes of hepatitis such as excessive alcohol consumption (>140 g/week) were diagnosed with NAFLD [28,29].

### Serum total testosterone

Blood samples for the measurement of serum total testosterone were collected *via* venipuncture performed between 8:00 and 11:00 AM. Serum total testosterone was measured using the Coat-A-Count total testosterone kit (Siemens Diagnostics Inc., Los Angeles, CA, USA). The Coat-A-Count procedure is a solid-phase radioimmunoassay that uses a testosterone-specific antibody-coated polypropylene tube and reports results within the range of 0.04–16 ng/mL.

### Other clinical and laboratory assessments

Medical history and life style, including information on liver disease, diabetes, smoking, alcohol consumption, and exercise, were documented using self-report questionnaires. We surveyed the average frequency and amount of alcohol consumption per week. Anthropometric data were measured by trained personnel, who used a standardized protocol and instruments. Height and body weight were measured using a digital scale, and BMI (kg/m^2^) was calculated. Waist circumference (WC) was measured at the midpoint between the lower costal margin and the iliac crest [30]. Laboratory examinations included determination of AST, ALT, gamma-glutamyl transpeptidase (γ-GT), alkaline phosphatase (ALP), total bilirubin, fasting glucose, fasting insulin, glycosylated hemoglobin (HbA1c), high-sensitivity C-reactive protein (hs-CRP), total cholesterol, and triglyceride (TG) levels. Venous blood samples were taken from all examinees between 8:00 and 11:00 AM after a minimum 14-h overnight fast. All biochemical determinations were conducted in the same laboratory with standard methods. Homeostasis model assessment of insulin resistance (HOMA-IR) was used as an indicator of insulin resistance and was defined as follows: fasting insulin (μIU/mL) × fasting plasma glucose (mmol/L)/22.5 [31]. Data on adipose tissue area were acquired through use of the CT cross-sectional scan, a validated procedure that has been demonstrated to show very low interobserver variation [32,33]. The subjects underwent abdominal CT with a 16-detector row CT scanner (Somatom Sensation 16; Siemens Medical Solutions, Forchheim, Germany) in the supine position. A 5-mm-thick slice obtained at the level of the umbilicus with a 0.5-s scan time was used to calculate abdominal fat compartments by means of CT (Rapidia 2.8; INFINITT, Seoul, Korea). VAT was defined as intraperitoneal fat bounded by the parietal peritoneum or transversalis fascia, excluding the vertebral column and paraspinal muscles, organs, blood vessels, and bowels.

### Statistical analysis

The general characteristics of the participants grouped according to the presence of NAFLD were compared using the Student’s *t*-test, the two-sample Wilcoxon rank-sum test, and Pearson’s Chi-squared test. We constructed 3 models and used the multiple logistic regression to explore the association of serum total testosterone with NAFLD, after adjusting for variables such as age, diabetes, smoking, exercise, BMI, VAT, HOMA-IR, and hs-CRP. The statistical significance of the nonlinear effects of independent variables was tested by the likelihood ratio test in the logistic regression models. Multicollinearity was assessed using the variance inflation factors (VIFs) [34]. A large proportion of our study subjects had normal serum total testosterone levels over 3 ng/mL. Therefore, we additionally analyzed the association between NAFLD and serum total testosterone in the subgroup of 413 subjects with normal serum total testosterone levels [35]. The statistical analyses were performed using STATA, version 10.0. and R version 2.12.2 (The R Foundation for Statistical Computing, Vienna, Austria). Statistical significance was defined by a two-tailed *p*-value of <0.05.

## Results

### General characteristics of the study subjects

NAFLD was found in 251 subjects (50.71%). Men with NAFLD showed lower testosterone levels (3.94 ng/mL *vs.* 4.83 ng/mL) and more unfavorable metabolic profiles. The mean VAT in the NAFLD group was significantly higher than that in the non-NAFLD group (164.79 cm^2^*vs.* 106.74 cm^2^). The NAFLD group had higher proportions of diabetics (27.09% *vs.* 12.30%), and elevated FBS levels (101 mg/dL *vs.* 96 mg/dL). The NAFLD group also showed significantly higher hs-CRP (0.09 mg/dL *vs.* 0.04 mg/dL) and HOMA-IR (2.67 mg/dL vs 1.76 mg/dL) than the non-NAFLD group. Further characteristics of study subjects with and without NAFLD are presented in Table 1.

**Table 1:** General characteristics of study subjects categorized by the presence of NAFLD

	**no NAFLD (n = 244)**	**NAFLD (n = 251)**	***p* value**
Age *	54.98 (10.22)	53.85 (9.83)	0.2118
Total testosterone (ng/mL)	4.83 (3.73–5.96)	3.94 (3.10–4.85)	< 0.0001
Smoking †			0.310
non-smoker	72 (29.51)	59 (23.51)	
ex-smoker	110 (45.08)	125 (49.8)	
current smoker	62 (25.41)	67 (26.69)	
drinking amount (g/week)	21.04 (0–71.01)	8.53 (0–59.7)	0.0595
Exercise †			0.013
yes	193 (79.1)	174 (69.32)	
WC (cm)	85 (81–89.9)	91.5 (88–95.5)	< 0.0001
BMI (kg/m^2^)	23.56 (22.13–25.10)	26.14 (24.38–27.78)	< 0.0001
VAT (cm^2^)	106.74 (80.62–142.86)	164.79 (135.97–197.95)	< 0.0001
Diabetes †			< 0.001
yes	30 (12.30)	68 (27.09)	
FBS (mg/dL)	96 (90–103)	101 (93–114)	< 0.0001
SBP (mmHg) *	118.97 (13.02)	122.44 (13.86)	0.0043
DBP (mmHg) *	79.15 (9.81)	80.83 (10.94)	0.0734
total cholesterol (mg/dL) *	196.27 (34.46)	199.79 (37.10)	0.2747
TGs (mg/dL)	95 (71–130)	140 (99–195)	< 0.0001
HDL-C (mg/dL)	51 (44–59)	44 (40–51)	< 0.0001
LDL-C (mg/dL) *	125.51 (33.43)	130.18 (33.26)	0.1198
Total bilirubin (mg/dL)	1.1 (0.85–1.3)	1.1 (0.9–1.3)	0.687
ALP (IU/L)	59 (49.5–69)	62 (52–72)	0.018
AST (IU/L)	22 (19–27)	26 (22–33)	< 0.0001
ALT (IU/L)	22 (17–30)	33 (24–49)	< 0.0001
γ-GT (IU/L)	27 (20–39)	34 (26–49)	< 0.0001
hs-CRP (mg/dL)	0.04 (0.04–0.15)	0.09 (0.03–0.2)	0.0001
HOMA-IR (mg/dL)	1.76 (1.33–2.29)	2.67 (1.96–3.48)	0.0001
Metabolic syndrome †			< 0.001
yes	41 (16.80)	130 (51.79)	

^a^ Data for continuous variables without a normal distribution are presented as the median (range, Quartile 25–Q75) and were tested by the two-sample Wilcoxon rank-sum test.

^b^ Data for continuous variables with a normal distribution are shown as the mean (± standard deviation) and were tested by the Student’s *t*-test.

^c^ In the case of categorized variables, the number (n) and proportion (%) of subjects were tested by the Pearson’s Chi-square test.

* Mean (± standard deviation).

† the number (n) and proportion (%) of subjects.

*WC*, waist circumference; *BMI*, body mass index; *VAT*, visceral adipose tissue; *FBS*, fasting blood sugar; *SBP*, systolic blood pressure; *DBP*, diastolic blood pressure; *TGs*, triglycerides; *HDL-C*, high-density lipoprotein cholesterol; *LDL-C*, low-density lipoprotein cholesterol; *ALP*, alkaline phosphatase; *AST*, aspartate aminotransferase; *ALT*, alanine aminotransferase; *γ-GT*, gamma-glutamyl transpeptidase; *hs-CRP*, high-sensitivity C-reactive protein; *HOMA-IR*, homeostasis model assessment of insulin resistance.

### NAFLD and serum testosterone levels

**Figure 1:**
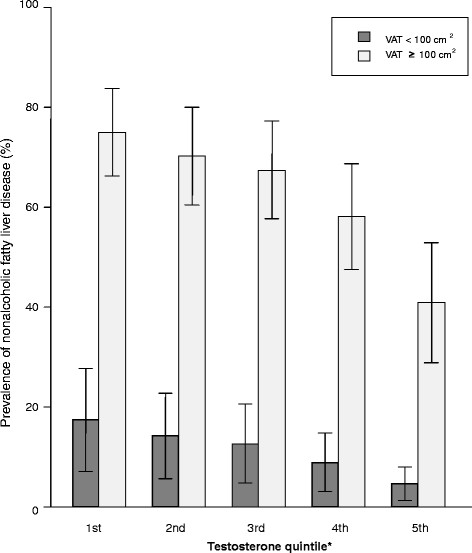
**Age-adjusted prevalence of non-alcoholic fatty liver disease by serum testosterone levels (stratified by VAT)**^**a**^**the range of serum testosterone quintiles (ng/mL): 1st quintile (0.11–3.17), 2nd quintile (3.18–3.88), 3rd quintile (3.92–4.78), 4th quintile (4.8–5.7), and 5th quintile (5.71–13.43) VAT, visceral adipose tissue.**

In both high- (VAT ≥ 100 cm^2^) and low- (VAT < 100 cm^2^) VAT groups, the mean age-adjusted prevalence of NAFLD decreased as the serum testosterone level increased from the 1^st^ quintile to the 5^th^ quintile (Figure 1). The association between serum total testosterone levels and NAFLD was examined by employing multiple logistic regression analysis with potential confounding variables such as age, smoking, diabetes, exercise, BMI, TGs, HDL-C, HOMA-IR, hs-CRP, and VAT controlled. An inverse relationship between serum total testosterone levels and NAFLD was shown in all models (*p* value: 0.0004 in model 1, 0.0006 in model 2, and 0.004 in model 3) (Table 2). As the serum testosterone level decreased from the 5^th^ quintile to the 1^st^ quintile, the adjusted odds ratio (OR) for NAFLD increased. The OR and 95% CI values in the 1^st^ quintile were 5.12 (CI, 2.43–10.77) in model 1, 4.99 (2.36–10.57) in model 2, and 4.52 (2.09–9.80) in model 3. A linear trend was seen in all models (*p* value for the trend < 0.001 for all models). Although the association was slightly attenuated in models 2 and 3 compared to those in model 1, the statistical significance of the association between serum total testosterone level and NAFLD persisted. The correlation coefficients between VAT and BMI, TGs, HDL-C, HOMA-IR, and hs-CRP were 0.653, 0.300, -0.312, 0.205, and 0.147, respectively. However, the VIFs for independent variables in all models in Table 2 were lower than 2.1. Therefore, no evidence of a serious multicollinearity effect was present. Similar results were observed in the subgroup analysis conducted with 413 subjects who had normal serum testosterone levels (≥3 ng/mL). An inverse association between serum total testosterone levels and NAFLD was also shown in all models (*p* value: 0.0036 in model 1, 0.0044 in model 2, and 0.0292 in model 3). All models showed a linear trend (*p* value for the trend: 0.001 in model 1 and model 2; 0.004 in model 3) Additional file 1: Table S1.

**Table 2:** The association between NAFLD and serum testosterone levels in the 495 subjects included in the study

	Model 1*		**Model 2†**		**Model 3‡**	
**Total testosterone (ng/mL)**	**OR (95% CI)**	***p* value**	**OR (95% CI)**	***p* value**	**OR (95% CI)**	***p* value**
1st quintile (0.11–3.17),	5.12 (2.43–10.77)	0.0004	4.99 (2.36–10.57)	0.0006	4.52 (2.09–9.80)	0.0040
2nd quintile (3.18–3.88)	2.62 (1.28–5.38)		2.48 (1.20–5.12)		2.31 (1.09–4.88)	
3rd quintile (3.92–4.78)	3.39 (1.68–6.84)		3.33 (1.65–6.75)		2.72 (1.31–5.65)	
4th quintile (4.80–5.70)	2.30 (1.12–4.70)		2.22 (1.09–4.57)		2.01 (0.96–4.23)	
5th quintile (5.71–13.43)	1		1		1	
*p* for the trend	< 0.001		< 0.001		< 0.001	

^a^ Multiple logistic regression was used to analyze the associations between NAFLD and serum total testosterone levels.

^b^*p* value: tested by the Wald test.

* Model 1: adjusted for age, smoking, diabetes, exercise, BMI, TGs, and HDL-C.

† Model 2: Model 1 + HOMA-IR and hs-CRP.

‡ Model 3: Model 2 + VAT (as the continuous variable).

*OR*, odds ratio; *CI*, confidence interval.

## Discussion

In this study, we found that low levels of serum total testosterone was independently associated with NAFLD among apparently healthy Korean men. This inverse association between serum testosterone and NAFLD persisted even after controlling for the effect of VAT, insulin resistance (as HOMA-IR), and low-grade inflammation (as hs-CRP), which are considered as possible links between testosterone and NAFLD.

Few studies have investigated the association between testosterone and fatty liver, and even fewer have addressed the potential relation between testosterone and NAFLD. A case–control study on men with chronic alcoholism suggested no association between serum testosterone and fatty liver [36], but this study included only 20 cases and 20 age-matched controls, with the study population consisting of chronic alcoholics. By contrast, a recent large observational study on 1912 German men showed an inverse association between hepatic steatosis and serum testosterone levels [25], a finding consistent with that in our study.

The mechanisms underlying the association between testosterone and NAFLD are still largely unclear. One possible explanation is the accumulation of VAT and subsequent insulin resistance among men with low serum testosterone. Men with testosterone levels in the lower range are well known to have an increased risk of VAT accumulation, which in turn is related to higher insulin resistance [17,22,37]. Excess VAT results in exposure of the liver to higher amounts of free fatty acids, which lead to hepatic insulin resistance and ultimately to systemic insulin resistance [38], and which are involved in the pathogenesis and prognosis of NAFLD [15,16]. There is also evidence that testosterone directly improves insulin sensitivity [39]. Research conducted at the Massachusetts General Hospital concluded that impaired Leydig cell function is directly associated with insulin resistance in men [39]. These findings led to the assumption that the association between low serum total testosterone levels and NAFLD might be mediated by the effect of VAT and insulin resistance. In the same context, the attenuation of the association between serum testosterone and NAFLD after adjusting for VAT and HOMA-IR suggests that VAT and insulin resistance may partially influence the pathway between testosterone and NAFLD.

Low-grade inflammation could be another possible link between testosterone and NAFLD. The chronic inflammatory state is fundamental to the progression of NAFLD [40,41]. Low serum testosterone is associated with markers of inflammation [42-44], and testosterone replacement therapy in men has been observed to have an anti-inflammatory effect, with a reduction in TNF-α, IL-1b, and sIL-6r levels and an increase in IL-10 and hs-CRP levels [1,26,42,45,46].

Testosterone may also influence microRNAs (miRNAs) in the liver or the activity of hepatic lipase [6,44,47]. Recent studies provide evidence that miRNAs are abundant in the liver and modulate a diverse spectrum of liver functions [6]. Moreover, deregulation of miRNA expression may be a key pathogenetic factor in many liver diseases [6]. Testosterone has been reported to potentially regulate miRNAs in the mouse liver [44], but the molecular mechanisms underlying transcriptional regulation of miRNA genes in the liver remain largely unknown [6]. A study reported that the activity of hormone-sensitive lipase is affected by testosterone in the adipose tissue and heart of the male rat [47]. These observations have only been reported in animals; hence, we could not establish definite conclusions. Further studies are necessary to uncover new mechanisms linking testosterone and NAFLD.

Our study had some limitations. First, we used ultrasonography, which cannot identify fatty infiltration below 30%, as the mode of diagnosis. While liver biopsy is regarded as the gold standard for detecting hepatic steatosis [2], liver biopsy was not feasible in the case of this population-based study. Ultrasonography is the diagnostic test of choice for NAFLD, because it is noninvasive, safe, sensitive (up to 93%), and specific (up to 89%) in terms of identifying fatty infiltration [48]. Its clinical efficacy in this regard has also been proven, since a recent study on Koreans showed that ultrasonography-diagnosed NAFLD acts as an independent risk factor for coronary heart disease [11]. Second, although we checked the medical history of liver disease and previous use of steatogenic drugs through the self-report questionnaires, it was possible that rare liver diseases such as autoimmune hepatitis and Wilson’s disease and fatty liver disease caused by steatogenic drugs were not completely excluded considering the recall bias of self-report questionnaires. Third, only serum total testosterone was used to assess testosterone levels because of the absence of available data on free testosterone and sex-hormone binding globulin (SHBG). Fourth, the use of a convenience sample and a cross-sectional design limited the drawing of conclusions on causality. Fifth, the findings are restricted by sex because only men were used in this study. Since testosterone plays a role in energy metabolism in females as well, the role of this hormone in female NAFLD will also need to be examined. Despite these limitations, the contribution of this study is that it is the first one to identify the association of testosterone with NAFLD after adjusting for the effect of VAT and insulin resistance. Further longitudinal cohort studies are needed to verify the causal relationship between NAFLD and total serum testosterone levels, and well-designed randomized clinical trials could show whether testosterone replacement therapy can improve NAFLD.

## Conclusions

In conclusion this study shows that low levels of serum total testosterone was independently associated with NAFLD. This inverse association was independent of the effect of VAT, insulin resistance (as HOMA-IR), and low-grade inflammation (as hs-CRP).

## Abbreviations

NAFLD, Nonalcoholic fatty liver disease; CVD, Cardiovascular disease; VAT, Visceral adipose tissue; BMI, Body mass index; CT, Computed tomography; HBsAg, Hepatitis B surface antigen; Anti-HCV, Hepatitis C antibody; WC, Waist circumference; AST, Aspartate aminotransferase; ALT, Alanine aminotransferase; γ-GT, Gamma-glutamyl transpeptidase; ALP, Alkaline phosphatase; HbA1c, Glycosylated hemoglobin; TGs, Triglycerides; HDL-C, High-density-lipoprotein cholesterol; HOMA-IR, Homeostasis model assessment of insulin resistance; hs-CRP, High-sensitivity C-reactive protein; OR, Odds ratio; VIFs, Variance inflation factors.

## Competing interest

The authors have no conflicts of interest to declare.

## Authors’ contributions

SK contributed to the design of the study, performed the statistical analysis, drafted the manuscript, and revised it in accordance with suggestions from the other authors. HK contributed to the study conception and design, data collection, analysis and interpretation of data, participated in the critical revision of the manuscript for important intellectual content. JHP contributed to the design of the study, interpretation of data, participated in the critical revision of the manuscript for important intellectual content. BC contributed to design of the study, interpretation of data, participated in the critical revision of the manuscript for important intellectual content. DK participated in data collection, analysis and interpretation of data, participated in the critical revision of the manuscript for important intellectual content. SWO participated in data collection, analysis and interpretation of data, participated in the critical revision of the manuscript for important intellectual content. CML participated in data collection, analysis and interpretation of data, participated in the critical revision of the manuscript for important intellectual content. HCC participated in the data collection, analysis and interpretation of data. All authors read and approved the final manuscript.

## Funding

This research did not receive any specific grant from any funding agency in the public, commercial, or not-for-profit sector.

## Pre-publication history

The pre-publication history for this paper can be accessed here:

http://www.biomedcentral.com/1471-230X/12/69/prepub

## Supplementary Material

Additional file 1: **Table S1.** The association between NAFLD and serum total testosterone levels (in the subgroup of 413 subjects with total testosterone ≥ 3.0 ng/mL). Click here for file
