# Supplementary material for: A low level of serum total testosterone is independently associated with nonalcoholic fatty liver disease
Source: BMC Gastroenterol. 2012 Jun 12;12:69. doi: 10.1186/1471-230X-12-69 (PMC3406998; doi:10.1186/1471-230X-12-69)
Supplement: Additional file 1: — Table S1. The association between NAFLD and serum total testosterone levels (in the subgroup of 413 subjects with total testosterone ≥ 3.0 ng/mL). [file 1471-230X-12-69-S1.doc]

Additional Files

Supplementary Table 1

The association between NAFLD and serum total testosterone levels (in the subgroup of 413 subjects with total testosterone ≥ 3.0 ng/mL)

|  | Model 1* |  | Model 2† |  | Model 3‡ |  |
| --- | --- | --- | --- | --- | --- | --- |
| Total testosterone (ng/mL) | OR (95% CI) | *p* value | OR (95% CI) | *p* value | OR (95% CI) | *p* value |
| 1st quintile (3–3.67) | 3.63 (1.61–8.19) | 0.0036 | 3.59 (1.58–8.13) | 0.0044 | 3.20 (1.38–7.42) | 0.0292 |
| 2nd quintile (3.68–4.34) | 2.69 (1.22–5.92) |  | 2.54 (1.15–5.63) |  | 1.98 (0.86–4.53) |  |
| 3rd quintile (4.35–4.96) | 3.59 (1.61–8.00) |  | 3.56 (1.59–7.96) |  | 2.78 (1.21–6.39) |  |
| 4th quintile (4.97–5.94) | 1.49 (0.67–3.32) |  | 1.46 (0.65–3.27) |  | 1.32 (0.57–3.04) |  |
| 5th quintile (5.96–13.43) | 1 |  | 1 |  | 1 |  |
| *p* for the trend | 0.001 |  | 0.001 |  | 0.004 |  |

^a^ Multiple logistic regression was used to analyze the associations between NAFLD and serum total testosterone levels

^b^ *p* value: tested by the Wald test

* Model 1: adjusted for age, smoking, diabetes, exercise, BMI, TGs, and HDL-C

† Model 2: Model 1 + HOMA-IR and hs-CRP

‡ Model 3: Model 2 + VAT (as the continuous variable)

OR, odds ratio; CI, confidence interval
